# Reversible switching of PEDOT:PSS conductivity in the dielectric–conductive range through the redistribution of light-governing polymers†

**DOI:** 10.1039/c7ra12624g

**Published:** 2018-03-20

**Authors:** Y. Kalachyova, O. Guselnikova, P. Postnikov, P. Fitl, L. Lapcak, V. Svorcik, O. Lyutakov

**Affiliations:** Department of Solid State Engineering, University of Chemistry and Technology Prague 16628 Czech Republic lyutakoo@vscht.cz; Department of Technology of Organic Substances and Polymer Materials, Tomsk Polytechnic University Tomsk 634050 Russia postnikov@tpu.ru; Department of Physics and Measurements, University of Chemistry and Technology Prague 16628 Czech Republic; Central Laboratories, University of Chemistry and Technology Prague Czech Republic

## Abstract

One of the biggest challenges in the field of organic electronics is the creation of flexible, stretchable, and biofavorable materials. Here the simple and repeatable method for reversible writing/erasing of arbitrary conductive pattern in conductive polymer thin film is proposed. The copolymer azo-modified poly(3,4-ethylenedioxythiophene) polystyrene sulfonate (PEDOT:PSS) was synthesized to achieve reversible photo-induced local electrical switching in the insulator–semimetal range. The photoisomerization of the polymer was induced by grafting nitrobenzenediazonium tosylate to the PSS main chains. While the as-deposited PEDOT:PSS thin films showed good conductivity, the modification procedure generated polymer redistribution, resulting in an island-like PEDOT distribution and the loss of conductivity. Further local illumination (430 nm) led to the azo-isomerization redistribution of the polymer chains and the creation of a conductive pattern in the insulating polymer film. The created pattern could then be erased by illumination at a second wavelength (470 nm), which was attributed to induction of reverse azo-isomerization. In this way, the reversible writing/erasing of arbitrary conductive patterns in thin polymer films was realized.

## Abstract 2

Chemical modification of PEDOT:PSS allows grafting light-switchable moieties to PSS chains and light induced reversible tuning of materials conductivity in dielectric-semimetal range.

## Graphical abstract

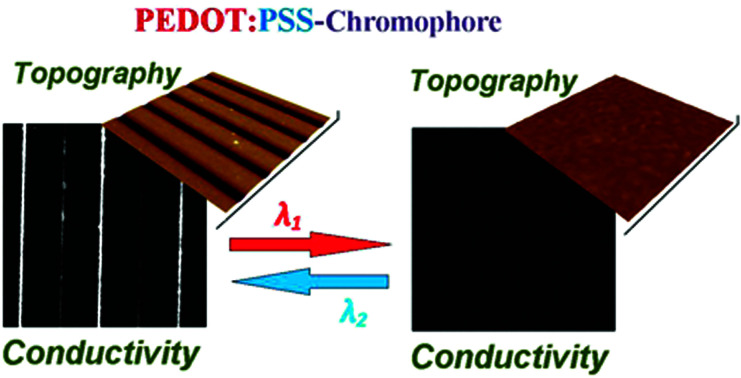

## Introduction

Flexible electronics present one of the most promising demands in the modern world. In this field, the family of conductive polymers (CPs) can be considered one of the most promising candidates.^1–7^ However, several accomplishments that have been made in the field of traditional semiconductor electronics remain incomplete for CPs. One of these is the local and reversible tuning of material properties by external stimuli.^8,9^ In the case of semiconductors, this led to great achievements, such as the development of magnetic (tuning of susceptibility) and optical memories (tuning of refractive index).^10,11^

Especially interesting is the creation of materials that can undergo local and reversible changes in conductivity with the possibility to write/erase the conductive paths *in situ*. The realization of this would open the door for the creation of new electronic devices and the implementation of unprecedented design principles.^12,13^ From this point of view, CPs are unique^14,15^ because their properties can be tuned in the conductive–dielectric range.^16,17^ Although the development of reversible writable/erasable electronics based on CPs has been expected, it has not yet been realized because of the problem of determining which stimuli should be applied. Traditional stimuli used for CPs primarily include chemical triggering, which is complicated to apply in practical situations.

Several attempts to create alternative, light triggerable conductivity of CPs have been reported.^18–20^ Typically, the chromophore molecules architecturally embedded in main chains of the CPs^21^ undergo reversible isomerization under illumination, leading to the disruption of molecular conjugation and decreased conductivity.^22–24^ However, the relatively low photo responses of these π-conjugated main chain-type polymers results in conductivity switching by only one order of magnitude.^25,26^ Better results have been achieved by preparing light-triggerable CPs in the form of nanofibers^27^ or by incorporating units with enhanced photo-switching response.^28^ However, to date, a significant increase in the available conductivity range has not been achieved, and the significant photo-switching of CP conductivity has not been realized.

Thus, despite the fact that CPs provide an extremely interesting platform for the creation of materials with light-switchable conductivity, only mediocre results (in terms of conductivity range) have been achieved in this field. This can be explained by the fact that previous works have focused on the incorporation of chromophores into the main polymer chain in attempts to achieve on/off functionality with conjugation-related electron pathways. Since the results achieved in this way have been far from ideal, we propose an alternative approach. In this work, we keep the structure of the CP unchanged but alter its distribution in the dielectric matrix from an island-like structure (non-conductive) to a fully connected (conductive) one. Based on this idea, the sufficient extension of the conductivity range under light illumination was successfully realized.

## Experimental

### Materials

Acetic acid (reagent grade, ≥99%), diethyl ether, deionized water, methanol (puriss. p.a., absolute, ≥99.8% (GC)), *p*-toluenesulfonic acid monohydrate (ACS reagent, ≥98.5%), *tert*-butyl nitrite (90%), 4-nitroaniline (≥99%), and PEDOT:PSS water suspension was supplied from Sigma-Aldrich. 4-Nitrobenzenediazonium tosylate was synthesized according to the published procedure.^29^

### Sample preparation

#### PEDOT:PSS preparation and modification

Glass slides serving as the sample backing were first cleaned by ultrasonic washing in deionized water, acetone, and isopropanol and subsequently dried. PEDOT:PSS films (thickness = *ca.* 300 nm) were deposited by spin-coating at 3000 rpm for 10 min. The polymer films were thermally annealed for 2 h at 100 °C under argon atmosphere. The dried PEDOT:PSS films were modified by immersing the samples in a 1 mM aqueous solution of 4-nitrobenzenediazonim tosylate for 20 min according the previously reported procedure.^30^ The PEDOT:PSS films were then washed by deionized water, methanol, and acetone and subsequently dried. Finally, the films were annealed for 2 h at 100 °C in argon atmosphere.

#### Laser-beam writing (LBW)

Surface pattering was accomplished by illumination with fiber coupled laser equipped with a fiber collimator and focused through a microscope objective (100× magnification). The laser had a wavelength of 430 nm and was operated in continuous mode with an output power of 5–15 mW. The speed of laser scanning was 50 μm s^−1^. To erase the created pattern, the full sample area was illuminated with laser light at 490 nm (laser power = 1.5 W, focused spot = 2 cm^2^, time of illumination = 3 h). To erase the created pattern, the full sample area was illuminated with a 490 nm laser (laser power = 1.5 W, focused spot = 2 cm^2^, time of illumination = 3 h).

#### Thermal annealing

The patterned PEDOT:PSS thin film was thermally annealed in air at 200 °C (±3 °C) for 3 h using a thermostat Binder oven. The heating rate was 5 °C min^−1^, and the annealed samples were left to cool to room temperature in air.

### Sample characterization

UV-Vis spectra were recorded using a Lambda 25 spectrometer (Perkin-Elmer) in the wavelength range of 300–600 nm. Raman scattering was measured on a Nicolet Almega XR Raman spectrometer (laser power = 15 mW) with an excitation wavelength of 785 nm. Each point of the surface was measured once with a 90 s accumulation time. ATR-IR spectra were measured using a Nicolet iS 10 FT-IR spectrometer.

Electrical sheet resistance was determined by a standard two-point technique using a KEITHLEY 487 pico-amperemeter. The electrical measurements were performed at a pressure of approximately 10 Pa to minimize the influence of atmospheric humidity. The typical error in the sheet resistance measurement did not exceed ±5%.

Scanning electron microscopy (SEM; LYRA3 GMU, Tescan, CR) and energy-dispersive spectroscopy (EDX; analyzer X-MaxN, 20 mm^2^ SDD detector, Oxford instruments) were used to study the morphologies and elemental distributions of the pristine, grafted, and patterned PEDOT:PSS. The samples were attached by carbon conductive tape to avoid sample charging. SEM-EDX measurements were carried out using an accelerating voltage of 10 kV.

X-ray photoelectron spectroscopy (XPS) was used to determine the surface chemical composition. The XPS spectra were recorded using an Omicron Nanotechnology ESCAProbeP spectrometer fitted with a monochromated Al K alpha X-ray source at 1486.7 eV. The analyzed area had dimensions of 2 × 3 mm^2^.

To characterize the sample surfaces and electrical maps, peak-force AFM was applied. Surface mapping was performed using an Icon (Bruker) on areas of 3 × 3 μm^2^ or 30 × 30 μm^2^. The measurements were performed in peak-force AFM mode using a Pt/Pd-coated tip with a constant voltage (3 V) and contact current value was evaluated.

## Results and discussion

**Fig. 1:**
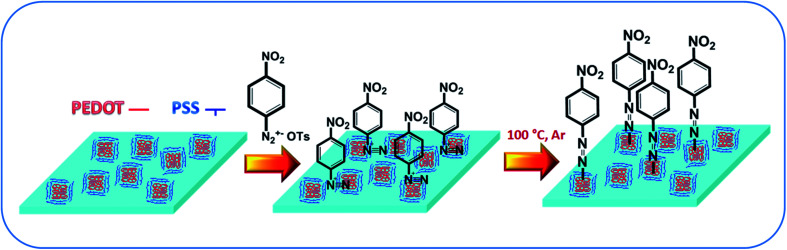
Schematic representation of PEDOT:PSS chemical modification.

A schematic representation of the modification procedure is given in Fig. 1. For the modification of PEDOT:PSS films, a two-step procedure was applied. In the first step, the simple immersion of the spin-coated films in a solution of arenediazonium tosylates led to the exchange of the diazo-cation and the formation of arenediazonium cation bonded to the SO_3_^−^ groups of the PSS chains. The excess arenediazonium tosylate was carefully removed by washing with deionized water. In the next step, the polymer films with chemisorbed diazo-cation were subjected to an annealing procedure followed by washing in organic solvents and drying in vacuum. The details of sample characterization, including elemental analysis and SEM-EDX data, are provided in the original paper.^30^ The presence of azo-bonds was confirmed by XPS and IR spectroscopies (Fig. S1–S3†). Briefly – after the grafting procedure, the nitrogen-related peaks appear on the XPS spectrum (Fig. S1†). Additionally, detailed investigation of XPS spectra indicates the presence of nitrogen in azo-form. In turn, an additional signal appears in the IR spectrum, which demonstrates the same kind of chemical transformation and confirms the proposed mechanism of grafting (Fig. S3†).

**Fig. 2:**
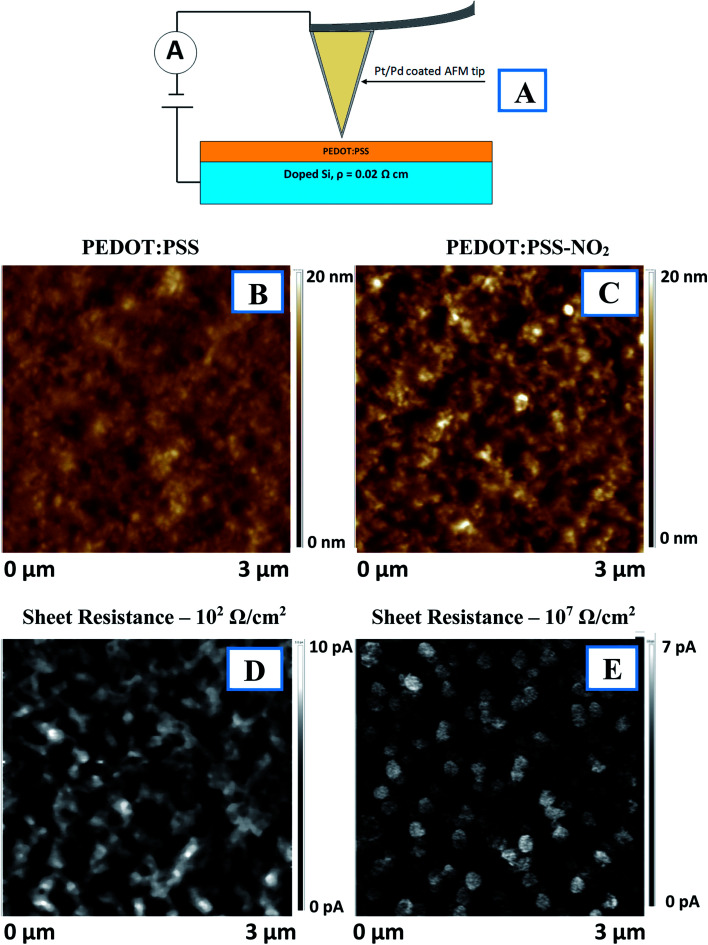
(A) Schematic representation of cAFM measurements. PEDOT:PSS thin film morphology before (B) and after (C) ADT–NO_2_ grafting. Distribution of the conductive area in PEDOT:PSS thin film before (D) and after (E) ADT–NO_2_ grafting.

The effect of PEDOT:PSS functionalization on thin-film conductivity was studied by measuring the sheet resistance using glass as a substrate and cAFM using doped silicon as a substrate (Fig. 2). Usually, the as-prepared PEDOT:PSS thin films exhibited island-like PSS domains surrounded by PEDOT chains.^31^ However, after chemical modification, the polymer distribution changed to an island-like distribution of PEDOT surrounded by PSS matrix (Fig. 2). PEDOT:PSS functionalization led to an increase in sheet resistance by several orders of magnitude (10^2^ Ω cm^−2^ before and 10^7^ Ω cm^−2^ after the procedure). The cAFM maps indicate that the decreased conductivity resulted from the redistribution of PEDOT and PSS in the film. While the pristine structure is a mutually penetrating 2D network of conductive area, functionalization led to its transformation into a separated island-like structure. As a result, the conductive pathways were disrupted, and the resistivity the PEDOT:PSS thin film apparently increased.

**Fig. 3:**
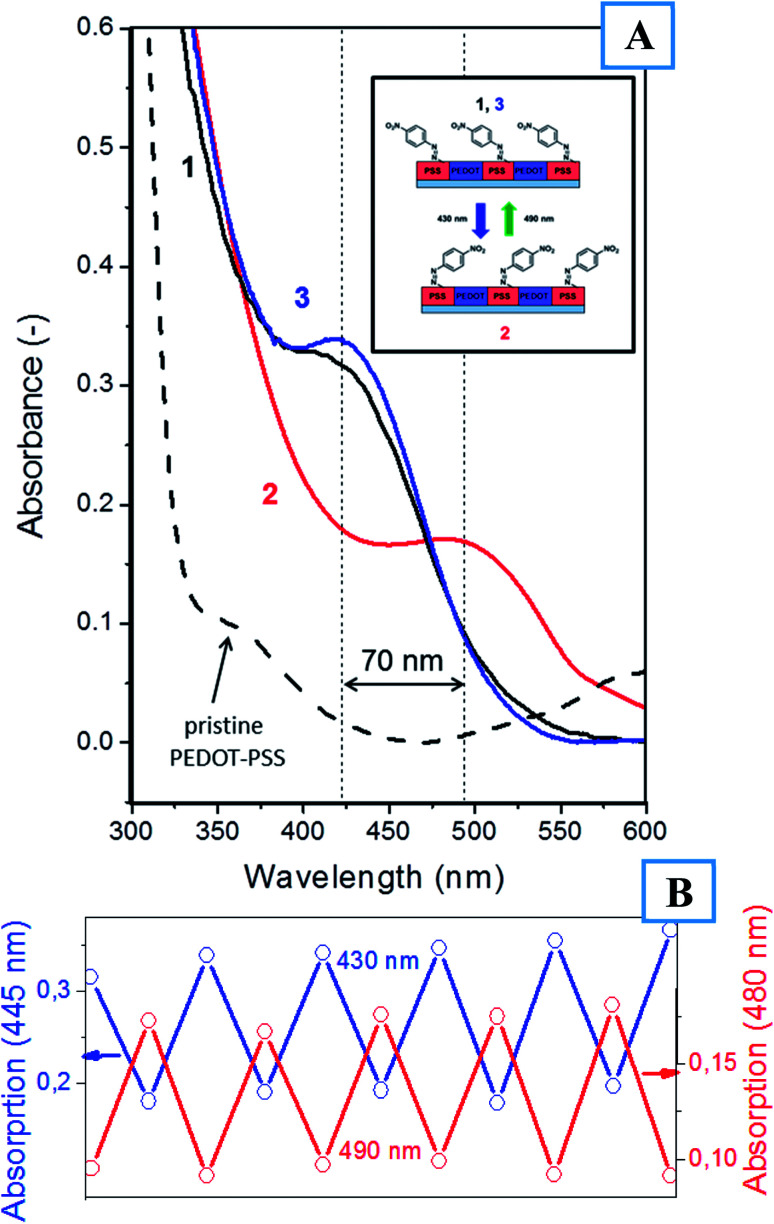
(A) UV-absorption spectra of a pristine PEDOT:PSS thin film and the film grafted with ADT–NO_2_ and subsequently illuminated at wavelengths of 405 and 532 nm. (B) Cyclic changes in PEDOT:PSS absorption coefficients upon successive irradiation at 430 and 490 nm.

The functionalization of PEDOT:PSS with azo-containing moieties must lead to significant changes in the film's optical properties. Fig. 3 presents the UV-Vis absorption spectra of PEDOT:PSS before and after functionalization. Functionalization led to an increase in film absorption and the appearance of a pronounced absorption band at 430 nm. This band is attributed to the *trans*-isomer of 4-(nitrophenyl)azo-groups^32^ and can undergo reversible *E*/*Z* isomerization under illumination at the corresponding wavelengths. Fig. 2 and 3 show that the surface modification of PEDOT:PSS led to a pronounced decrease in the conductivity of the polymer blend and the appearance of an optical absorption band located near 430 nm.

Azo-compounds are known to undergo light-triggerable *E*/*Z* photoisomerization, which is accompanied by a batochromic shift in the absorption peak. To verify this phenomenon, UV-Vis spectroscopy was implemented for samples illuminated at wavelengths of 430 and 490 nm (Fig. 3). As expected, illumination at 430 nm led to a shift in the absorption band to 495 nm resulting from the formation of the *Z*-isomers of (4-nitrophenyl)azo-groups. Subsequent light triggering at 490 nm led to the reversible formation of the starting *E*-isomer and to a shift in the absorption band to its pristine position (430 nm). This process was repeated several times, and the obtained results are shown in Fig. 3B. The optical *E*/*Z*-isomerization was found to be fully reversible, with the functionalized PEDOT:PSS repeatedly changing its properties over 10 cycles. Additional verification of the *E*/*Z* isomerization of the (4-nitrophenyl)azo-group was performed using IR spectroscopy. The presence of the N=N absorption peak (1430 cm^−1^) confirms the formation of *E*-isomer during PSS functionalization.^33^ After irradiation at 430 nm, this peak was shifted to 1592 cm^−1^ (–N=N– bond in the *Z*-isomer), validating the *E*/*Z*-isomerization process (Fig. S3†).^33^

The local irradiation of polymers containing azo-bonds results in a spatially separated isomerization process and the appearance of a concentration gradient of *E*/*Z* isomers, which in-turn initiates a material redistribution.^34,35^ In the PEDOT:PSS blend, only PSS contains diazo-containing moieties; thus, PSS is expected to be predominantly affected and redistributed under irradiation. However, the PSS flow also affects the PEDOT distribution and thus the arrangement of conductive areas. To introduce local *E*/*Z* isomerization and material redistribution, the LBW technique was applied. Optical microscopy of the structure created by LBW (Fig. S4†) confirms the material flow from the illuminated area and the creation of a pattern on the previously flat polymer surface.

The effect of laser intensity on the pattern formation was also studied, and the results are presented in Fig. S5.† As expected, the reduction in laser intensity led to the creation of less pronounced surface features. Further polymer heating (200 °C, 3 h) at ambient humidity resulted in the partial smoothing of the created pattern, although the effect of sample heating was weaker (Fig. S6†) than that of laser illumination. The effect of heating the azo-containing polymer was previously discussed.^36^ The azo-polymer was shown to have excellent heat stability, and created surface structure remained almost unchanged under heating up to 200 °C.

**Fig. 4:**
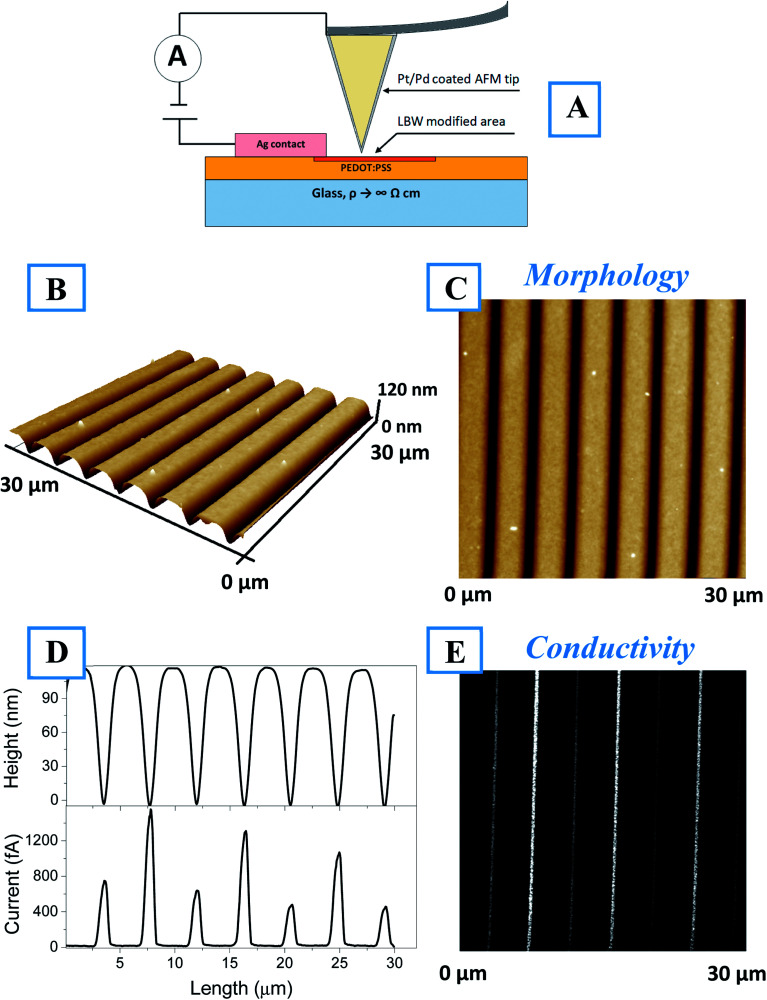
(A) Schematic representation of cAFM measurements. (B) 3D and (C) 2D surface morphology of the LBW-patterned thin film. (D) Comparison of the surface morphology profile and the conductivity profile and (E) the corresponding conductivity map.

The cAFM results presented in Fig. 4 show the surface morphology and conductivity. For better clarity, a schematic representation of the cAFM measurements, including the direction of electric current flow, is shown in Fig. 4A. Fig. 4B shows a 3D image of the surface morphology, from which a perfectly ordered array of lines created on the polymers surface is evident. The line geometry (*i.e.*, length and mutual distance) corresponds to the laser pathway on the polymer surface. Fig. 4C and D show the corresponding 2D morphologies and conductivity maps. It is evident that during polymer patterning, a conductive line array was created. The surface profiles taken along the morphology and corresponding scans are shown separately in Fig. 4E. Here, each valley on the morphology profile corresponds to conductive peak on the conductivity scan. The width of the conductive peak is smaller than the width of the morphology valley. Thus, the creation of conductive areas occurred only in the valley bottom. The AFM scan of single line, is also given in the Fig. S7(A–D),† as well as the possibility to create the conducting “twist” (Fig. S7E†).

**Fig. 5:**
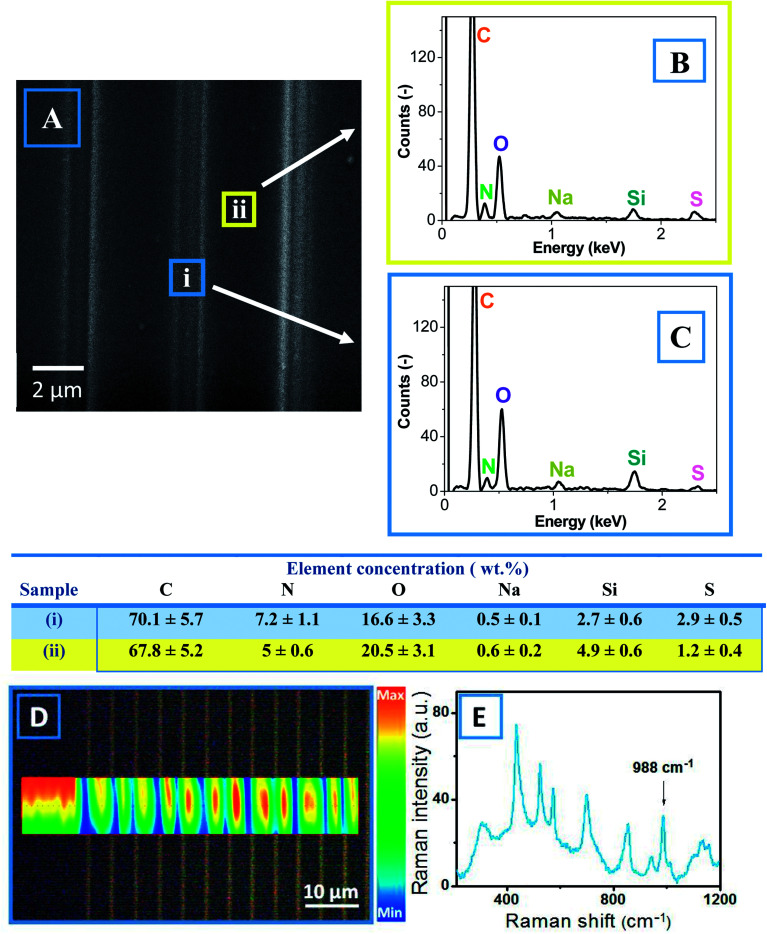
SEM-EDX images and Raman spectra of the created structures. (A) SEM image of a line array; the colored squares indicate the areas from which the EDX maps of elemental composition were collected. EDX maps of the valley area (B) and “top” area (C); the elemental compositions are given in the corresponding table. (D) Optical images of the patterned PEDOT:PSS surface and map of Raman response (988 cm^−1^ peak intensity distribution). (E) Typical Raman spectrum.

The creation of a conductive pattern resulting from PSS flow and the interconnection of PEDOT islands was further demonstrated by SEM-EDX and Raman measurements (Fig. 5). Based on the SEM scan of the patterned PEDOT:PSS surface, two places corresponding to the laser track and the unaffected surface were chosen and used to determine the elemental concentrations (Fig. 5A). The obtained EDX spectra are presented in Fig. 5B and C, and the concentrations of elements are given in the table below. The presence of characteristic peaks of carbon, oxygen, and sulfur are expected from the chemical composition of PEDOT:PSS. An additional peak of nitrogen appears due to PSS functionalization and can be considered as a marker of PSS content. To avoid error resulting from fluctuations in film thickness, the N/C ratio was taken as a parameter to characterize the PEDOT:PSS local composition. The N/C ratio in the valley was found to be smaller than that at the top. Thus, it can be concluded that during LBW, the PSS is pulled out, while the PEDOT remains in the laser-created valleys. From the Raman spectrum of PEDOT:PSS (see Fig. 5E), the characteristic Raman peak of PSS (SO_2_ stretch at 988 cm^−1^) was chosen, and its intensity deviation across the created line pattern is given in Fig. 5D. The measured Raman map (Fig. 5D) shows that the surface valleys (*i.e.*, laser-modified areas) contained lower amount of PSS, confirming that the PSS flowed away from the illuminated areas.

**Fig. 6:**
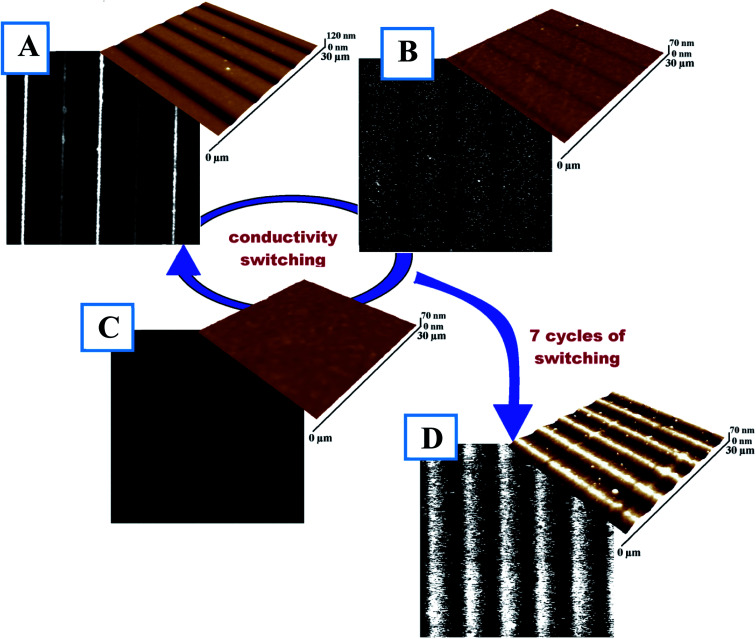
Conductivity maps and corresponding surface morphology for (A) modified and LBW-patterned (430 nm) PEDOT:PSS film; (B) film during pattern erasing (490 nm illumination, 30 min); (C) film with completely erased pattern (490 nm, 3 h); and (D) conductive line array prepared after several cycles of conductivity switching.

In the case of polymers containing diazo-moieties, isomerization reversibility can be used for reversible surface pattering/erasing. In our case, this phenomenon was also utilized for reversibly creating/erasing the conductive pattern in the thin PEDOT:PSS film. To erase the pattern, illumination at 490 nm was used. This wavelength returns the azo-bond into the *E*-configuration, eliminates the isomer concentration gradient, and initiates the “opposite” PSS flow. The reversible migration of PSS can be attributed to several possible mechanisms, including the thermal gradient, asymmetric diffusion-related forces, isomerization pressure, mean-field chromophore–light interaction, and gradients in permittivity or electric force.^37–42^Fig. 6 presents the full cycle of conductive pattern creation/erasing. After the first step (LBW), the well-ordered surface structure and corresponding conductivity pattern are created (Fig. 6A). Full sample illumination at 490 nm for 30 min (Fig. 6B) leads to substantial disappearance of the pattern with traces of the previous structure still present. Further illumination for 3 h results in complete surface flattening and the complete disappearance of conductivity paths. Thus, illumination at 490 nm induces inverse polymer flow and disrupts the conductive pathways. Moreover, the created flat surface can be used to record new conductive paths different from previous ones. The self-healing and return of the isomer to its previous state, which is typical in PEDOT:PSS, lead to the gradual erasing of the pattern accompanied by the return of PEDOT to it pristine, island-like structure and corresponding loss of conductivity in the thin polymer film. To check the cycling properties of the prepared structure, the writing/erasing process was repeated seven times, and results are presented in Fig. 6D. Here, the measured conductive patterns are not as perfect as in the initial cycles, probably because of material aging and degradation. Taking into account the higher exposition fluence required for pattern erasing, the presence of air humidity, and the potential heating of the polymer film by light exposure, the following complex mechanism of structure removal can be proposed: the laser-induced isomerization and heating in the presence of air moisture results in the smoothing out of the polymer structure and the return of PEDOT into its initial, island-like and non-conductive structure. We believe that this drawback can be overcome through further material tuning, optimization of polymer composition, changing of substituents in the azo-containing groups, and improvement of the grafting procedure and optical setup.

## Conclusion

A new revolutionary approach for fine tuning the conductivity of PEDOT:PSS films *via* laser-beam writing was suggested and verified. The procedure is based on the covalent grafting of (4-nitrophenyl)azo-chromophores to the PSS chains and the optical patterning of the polymers. During the chemical grafting of PSS, the redistribution of the PEDOT structure from a conductive interpenetrating network to a non-conductive, island-like structure occurs. The further local light-induced *E*/*Z*-isomerization of the attached (4-nitrophenyl)azo-groups leads to the spatial redistribution of the polymer blend and the creation of conductive pathways. Subsequent illumination of the PEDOT:PSS thin film at a second wavelength leads to the opposite chromophore isomerization, surface flattening and disruption of the previously created conductive paths. Particularly important is the fact that the redistribution process is reversible and allows for the repeating of writing/erasing cycles. The authors believe that the proposed approach can find application in the new generation of flexible, biodegradable and rewritable electronics. Moreover, it is expected that the writing/erasing procedure could be carried out through planar lithography with a mask in addition to through the LBW technique, facilitating the reversible creation/disruption of conductive patterns.

## Conflicts of interest

There are no conflicts to declare.

## Supplementary Material

RA-008-C7RA12624G-s001
