# Supplementary material for: Reversible switching of PEDOT:PSS conductivity in the dielectric–conductive range through the redistribution of light-governing polymers
Source: RSC Adv. 2018 Mar 20;8(20):11198–206. doi: 10.1039/c7ra12624g (PMC9078990; doi:10.1039/c7ra12624g)
Supplement: RA-008-C7RA12624G-s001 [file RA-008-C7RA12624G-s001.pdf]

Electronic Supplementary Material (ESI) for RSC Advances.
This journal is © The Royal Society of Chemistry 2018

Supplementary Information

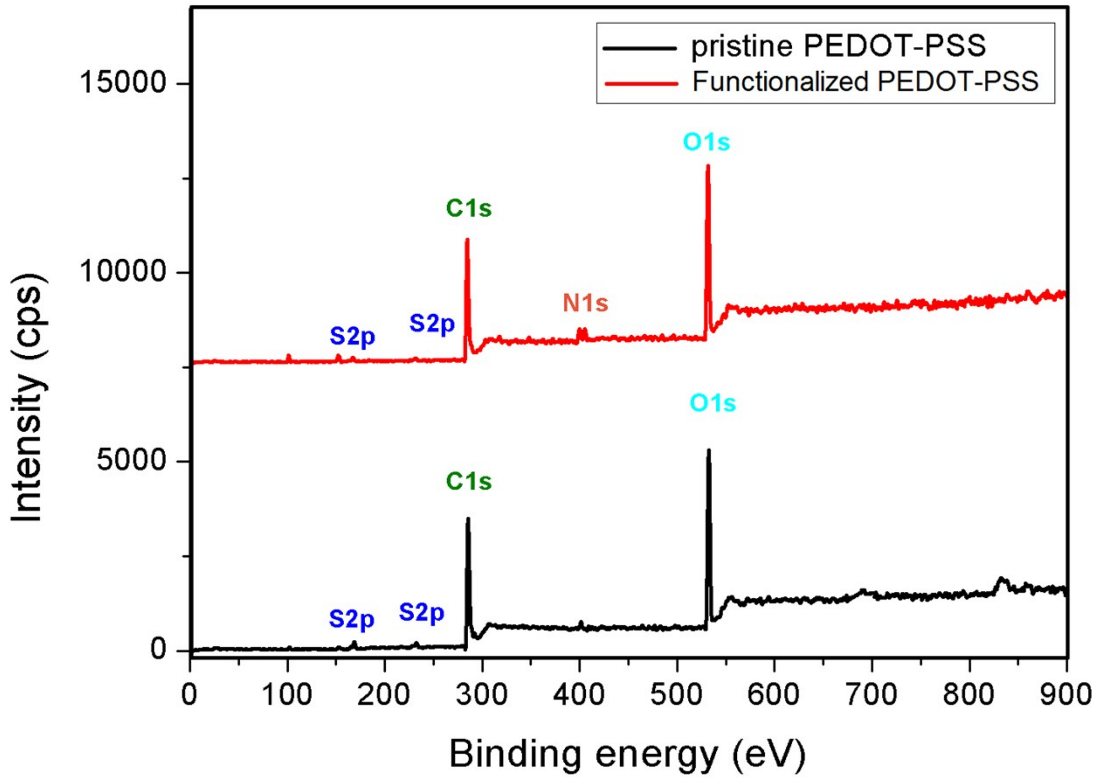

Fig. S1 XPS spectra of pristine and grafted PEDOT:PSS

Explanation: Before annealing two characteristic peak at 406.7 eV (NO_2 groups) and 401.6.7 eV, assigned to the presence of adsorbed diazo cations are observed. After annealing, there a peak at 401.2 eV appears which is assigned to azo-bonds, formed during thermally initiated grafting of ADT-NO_2.

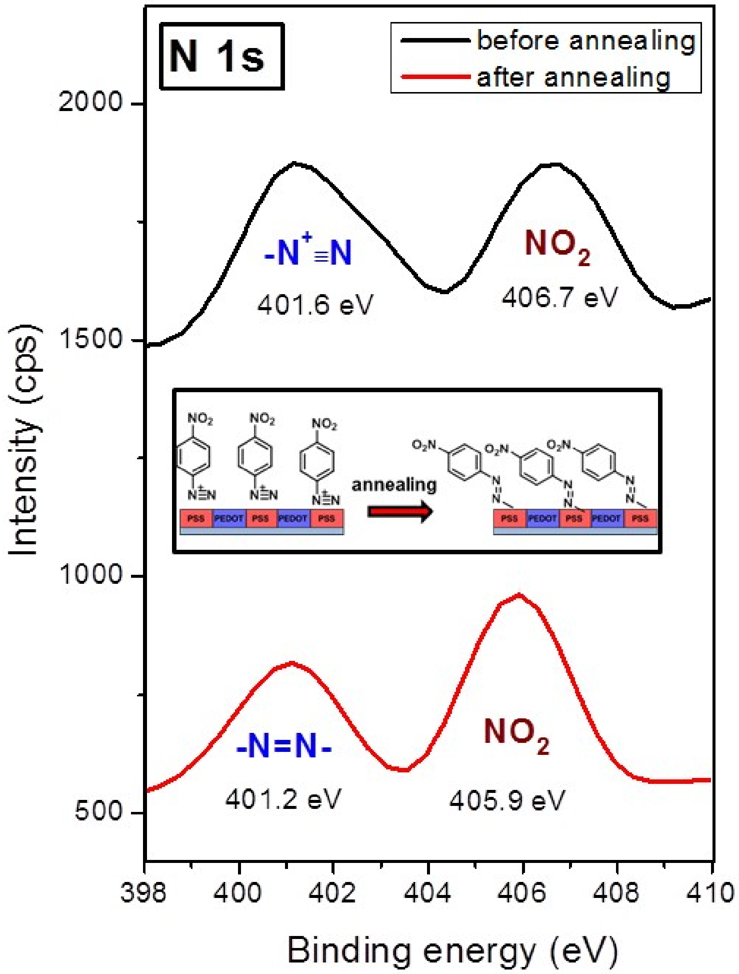

Fig. S2 Detailed depiction of nitrogen related XPS peaks.

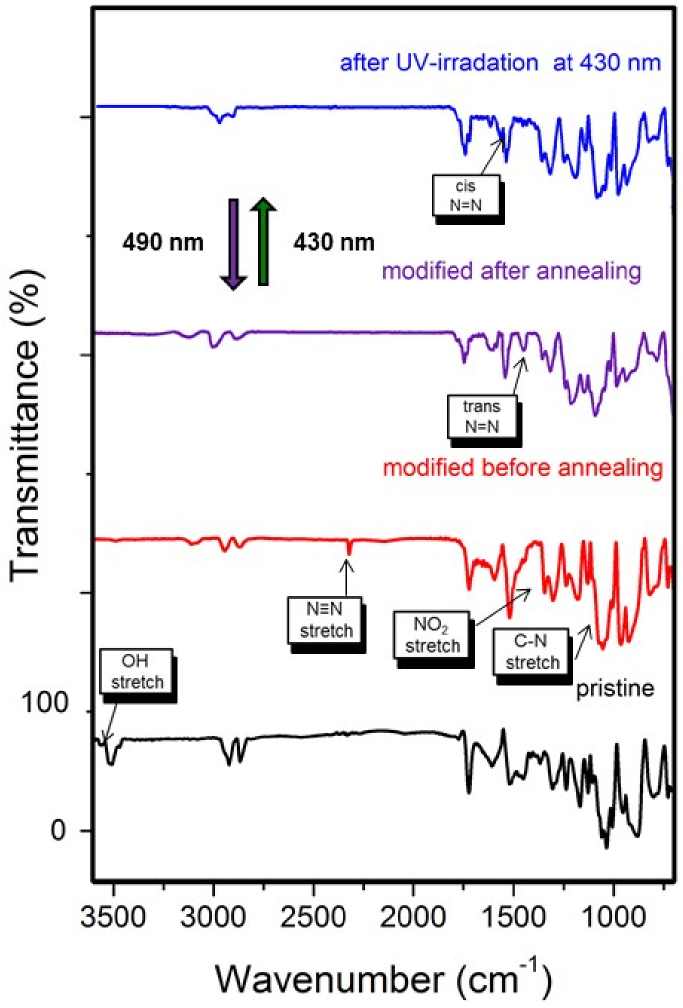

Fig. S3 ATR-IR transmission spectra of pristine PEDOT:PSS, PEDOT:PSS grafted with ADT-NO_2 before and after annealing procedure, and NO_2 grafted PEDOT:PSS after irradition with 405 nm wavelength.

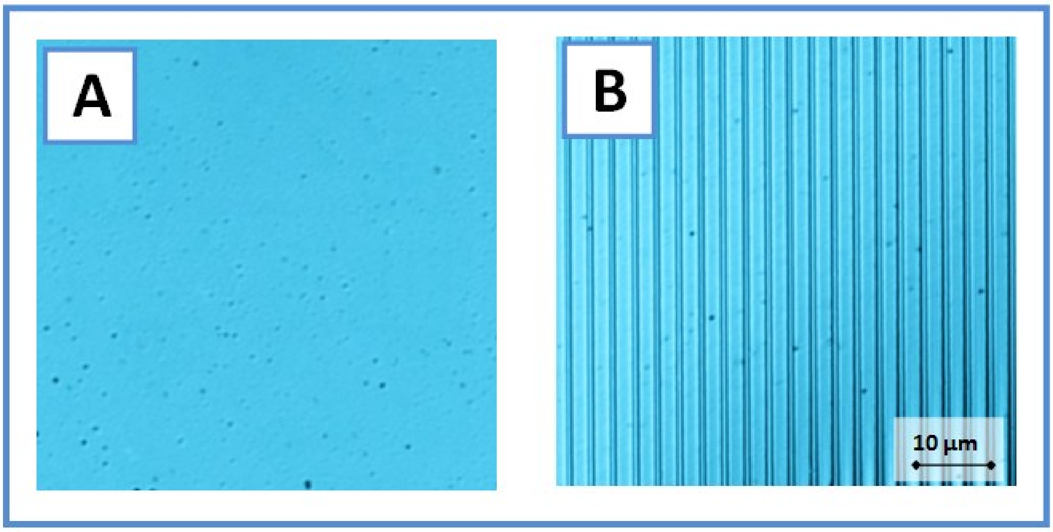

Fig. S4 The array of parallel lines, created on the PEDOT:PSS surface grafted with ADT-NO_2 by the LBW technique.

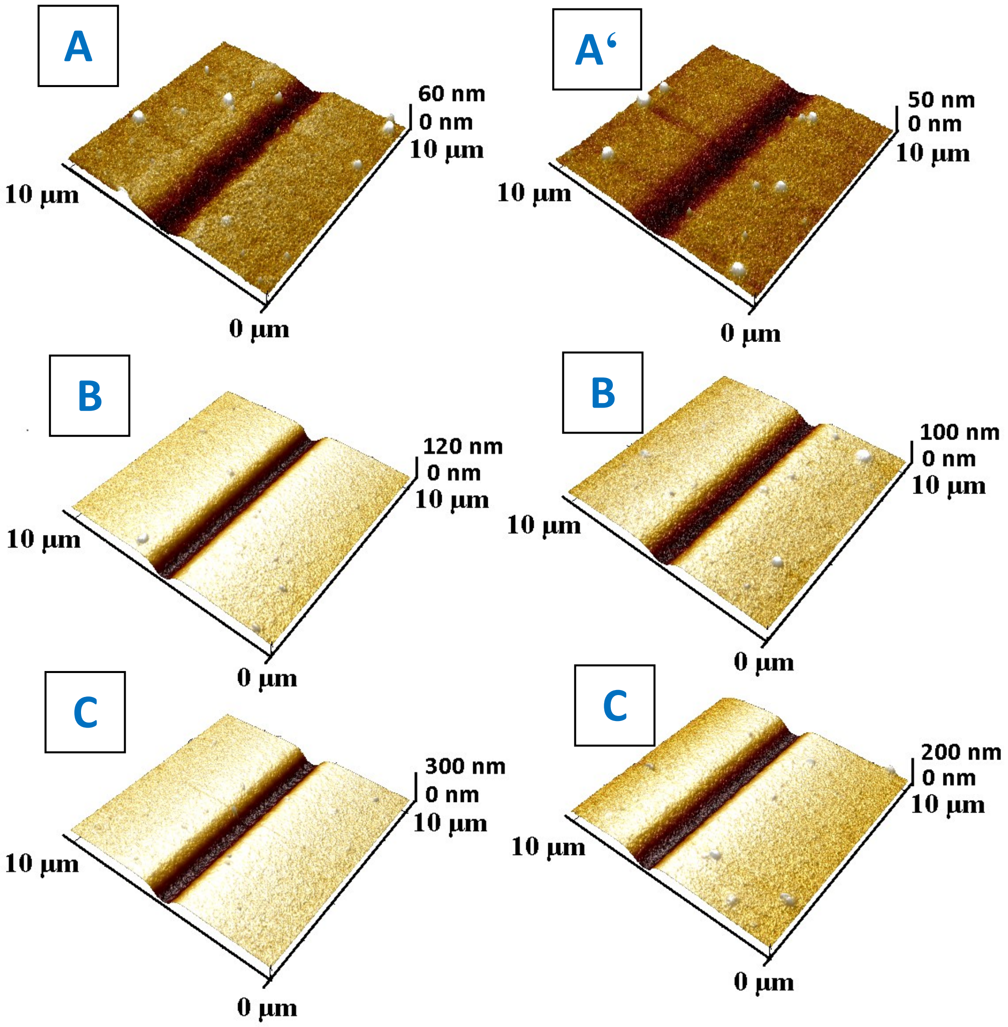

Fig. S5 Effect of laser power and subsequent thermal annealing on the profile of LBW created lines: A - 5mW; B - 10 mW; C - 15 mW. The left parts of the figure (A/, B/, C/) correspond to the same structures after thermal annealing.

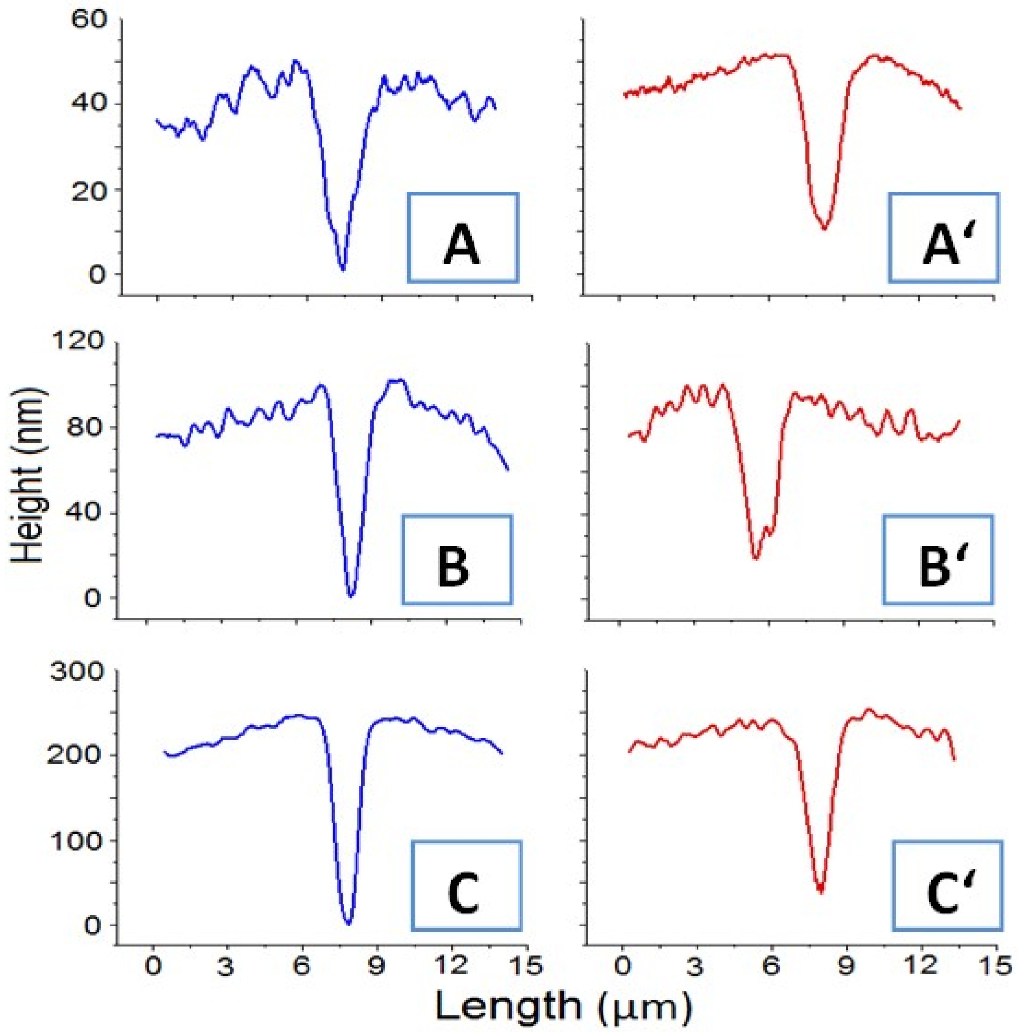

Fig. S6 Corresponding to the Fig. S5 surface profiles, taken before and after thermal annealing.

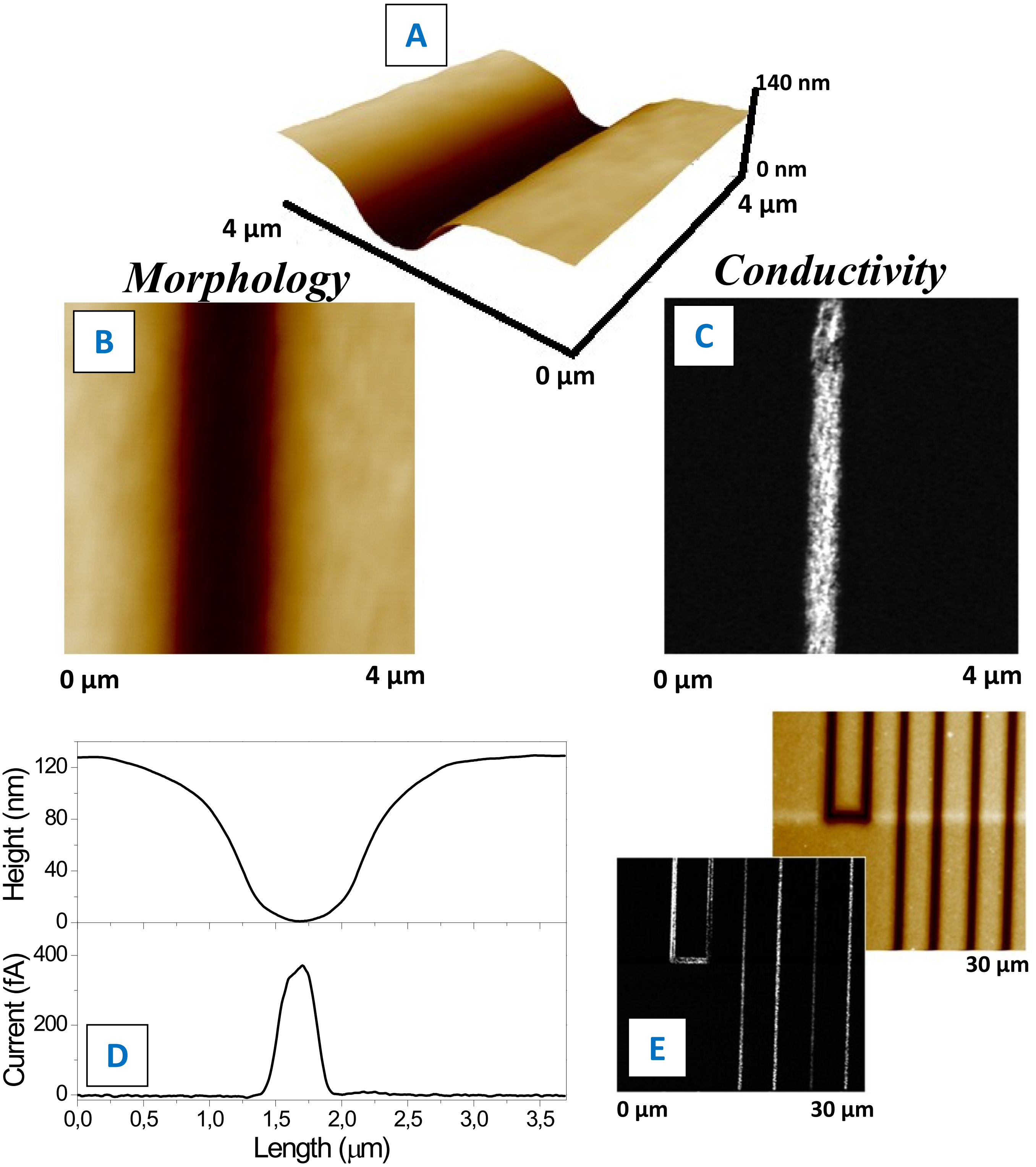

Fig. S7 Surface morphology and conductivity pattern created on the modified PEDOT:PSS surface. One single line from the Fig. 4 (see main article) measured at the higher resolution is shown.
